# Acute Stroke Management and Nursing Intervention

**DOI:** 10.7759/cureus.86820

**Published:** 2025-06-26

**Authors:** Chidinma S Madu, Victoria M Ajibade

**Affiliations:** 1 Neuro-Oncology, Duke University Health System, Durham, USA; 2 Pediatric Critical Care, Duke University Health System, Durham, USA

**Keywords:** acute stroke, dysphagia management, nursing interventions, patient education, stroke prevention, stroke rehabilitation, thrombolytic therapy

## Abstract

Stroke remains a leading cause of mortality and long-term disability worldwide, necessitating prompt and effective management strategies to optimize patient outcomes. Acute stroke management involves rapid diagnosis, timely pharmacological and mechanical interventions, and coordinated multidisciplinary care. Among healthcare professionals, nurses play a pivotal role in the early identification, stabilization, and ongoing management of stroke patients, particularly during the hyperacute and acute care phases. This review article explores current evidence-based approaches in acute stroke management, including the use of thrombolytics, thrombectomy, and neuroimaging techniques, while emphasizing the critical role of nursing interventions such as neurological assessment, airway and hemodynamic support, medication administration, and patient education. Furthermore, it discusses the integration of stroke care pathways and the impact of nurse-led initiatives on patient outcomes. This review, involving recent literature, highlights the importance of collaborative, guideline-driven care and the evolving responsibilities of nurses in improving the survival and recovery of patients experiencing acute stroke.

## Introduction and background

Acute stroke is a critical emergency that demands comprehensive, multidisciplinary care to minimize the risk of long-term disability and mortality [1]. Ischemic stroke is one of the leading causes of death and disability worldwide, accounting for the majority of all stroke cases. Therefore, the time taken in recognition, diagnosis, and initiation of treatment is inversely proportional to the outcomes for the patient. Nurses constitute an indispensable resource at every step of acute stroke management, from initial assessment and emergency interventions to monitoring and early rehabilitation. These duties are instrumental in having the nursing staff execute protocols for stroke care based on evidence, thus ensuring the timely delivery of treatment such as thrombolytic therapy while preventing any complications [2]. Nursing interventions, in addition to supporting medical treatment, also enhance recovery by catering to the physical, psychosocial, and educational needs of the patients and their families. This paper looks at the scope and impact of nursing care during the hyperacute and acute phases of stroke, with emphasis on evidence-based interventions, thrombolytic therapy, rehabilitation, and strategies to facilitate patient adherence to rehabilitation programs. 

## Review

Comprehensive review of evidence-based nursing interventions during the hyperacute and acute phases of stroke care

During the hyperacute and acute phases of stroke care, timely and evidence-based nursing interventions are critical for improving patient outcomes and minimizing the risk of long-term disability or death [3]. The hyperacute phase, typically defined as the first 24 hours following stroke onset, is a period where rapid assessment and intervention can significantly influence the course of treatment. Nursing care during this period focuses on early recognition of stroke symptoms, prompt activation of emergency response systems, and rapid initiation of diagnostic procedures such as neuroimaging to determine the type and severity of the stroke.

In the hyperacute phase, nurses play a pivotal role in ensuring that protocols for thrombolytic therapy, such as the administration of intravenous tissue plasminogen activator (tPA), are followed accurately and within the recommended time window [4]. This includes obtaining a thorough patient history, assessing contraindications, and closely monitoring vital signs and neurological status. Continuous neurological assessments using tools like the National Institutes of Health Stroke Scale (NIHSS) are essential for tracking changes in the patient’s condition and guiding treatment decisions [5]. In the acute phase, which spans from the first 24 hours up to several days post-stroke, nursing care shifts toward preventing complications and promoting neurological recovery. Nurses are responsible for maintaining airway patency, optimizing oxygenation, and ensuring hemodynamic stability, all of which are vital in preventing secondary brain injury. Blood glucose and temperature control are also critical components of nursing care, as hyperglycemia and fever have been associated with poorer stroke outcomes [6].

Moreover, nursing interventions during this phase include the prevention of common post-stroke complications such as aspiration pneumonia, deep vein thrombosis, and pressure injuries. This involves positioning techniques, early mobilization where appropriate, administration of prophylactic medications, and the use of assistive devices to maintain functional positioning [7]. Nurses also play a key role in supporting the initiation of rehabilitation therapies, coordinating with physical, occupational, and speech therapists to begin early interventions that support recovery.

Equally important is the emotional and psychological support provided by nurses. Patients and their families often experience significant distress and uncertainty following a stroke [8]. Nurses serve as educators and advocates, providing information about the condition, treatment options, and prognosis, while also addressing emotional needs through compassionate communication and supportive care [9].

Role of Nurses in Administration of Thrombolytic Therapy and Monitoring Outcomes

Nurses play an essential role in the administration of thrombolytic therapy and in monitoring patient outcomes following treatment, particularly in the context of ischemic stroke [10]. Their responsibilities begin with the rapid identification of eligible candidates for thrombolysis and extend through the meticulous monitoring required during and after administration of the therapy. The effectiveness of thrombolytic treatment, such as intravenous tPA, is highly time-dependent, and the nurse’s ability to act swiftly and accurately is crucial to patient recovery [11].

One of the primary responsibilities of nurses is to ensure that all eligibility criteria for thrombolytic therapy are met. This involves gathering a detailed and accurate patient history, including the time of symptom onset, current medications, and potential contraindications such as recent surgery, bleeding disorders, or uncontrolled hypertension. Nurses must also assist in facilitating immediate neuroimaging and laboratory assessments required to confirm an ischemic stroke diagnosis and rule out hemorrhagic stroke, which would contraindicate thrombolysis [12].

Once the decision to administer thrombolytic therapy is made, nurses are responsible for preparing and administering the medication in accordance with established protocols [13]. This includes verifying dosages based on patient weight, ensuring proper infusion techniques, and maintaining sterility and accuracy throughout the process. During the infusion and for at least 24 hours afterward, nurses provide continuous monitoring of neurological status, vital signs, and signs of potential complications such as intracerebral hemorrhage, angioedema, or systemic bleeding.

Neurological assessments are typically performed at frequent intervals using standardized tools such as the NIHSS to detect even subtle changes in the patient’s condition [14]. Nurses must be prepared to respond rapidly to any signs of deterioration by notifying the stroke team and initiating emergency protocols. In addition, they monitor blood pressure closely, as elevated or fluctuating levels can increase the risk of bleeding [15]. Strict blood pressure management protocols are often implemented, and nurses play a critical role in administering antihypertensive agents when needed.

Beyond the acute management phase, nurses also evaluate the effectiveness of thrombolytic therapy by documenting improvements or worsening of neurological function [16]. They collaborate with multidisciplinary teams to determine the next steps in care, whether it be intensive monitoring in a stroke unit or transition to rehabilitation services. They also educate patients and families about the treatment received, potential side effects, and the importance of adhering to follow-up care and secondary prevention strategies.

Nursing strategies for post-stroke rehabilitation

Nurse-led interventions play a vital role in enhancing stroke rehabilitation and recovery, with growing evidence supporting their effectiveness in improving patient outcomes [17]. These interventions focus on early mobilization, patient education, medication adherence, and coordination of multidisciplinary care, all of which contribute to faster functional recovery and reduced hospital readmissions.

Nurses are often at the forefront of initiating and maintaining rehabilitation activities, such as assisting with physical exercises, promoting self-care, and encouraging communication in patients with speech deficits. Their consistent engagement helps reinforce therapy goals and supports continuity of care. Moreover, nurse-led education programs have been shown to improve the understanding of stroke, lifestyle modifications, and risk factor management in patients and caregivers, which are critical for secondary prevention.

Studies have also demonstrated that nurse-led follow-up care, including home visits and telehealth check-ins, leads to higher satisfaction rates and better adherence to rehabilitation plans [18]. These interventions help identify complications early, address barriers to recovery, and provide psychological support, all contributing to better functional and emotional outcomes.

Review of Nursing Strategies to Improve Patient Adherence to Rehabilitation Programs Post-Stroke

Improving patient adherence to rehabilitation programs after a stroke is a critical component of nursing care, as consistent participation in therapy directly impacts recovery and long-term outcomes [19]. Nurses employ a range of strategies to support and enhance adherence, focusing on education, motivation, individualized care, and ongoing support [20]. One of the primary nursing strategies is patient and caregiver education. Nurses ensure that patients understand the purpose and benefits of rehabilitation, the expected timeline for recovery, and the risks associated with non-adherence [21]. Clear communication, using simple language and visual aids when necessary, helps bridge knowledge gaps and sets realistic expectations.

Motivational interviewing is another effective approach used by nurses to address ambivalence and build patient commitment to therapy. By exploring personal goals and values, nurses can help patients connect the rehabilitation process to meaningful life outcomes, which can increase intrinsic motivation [22].

Individualized care plans tailored to each patient’s physical, emotional, and social needs are also key [23]. Nurses assess barriers such as transportation issues, fatigue, depression, or cognitive impairments and work to find practical solutions or refer patients to appropriate support services. Involving caregivers in planning and decision-making further reinforces adherence by fostering a supportive environment. Regular follow-up and consistent encouragement are vital. Nurses often coordinate home visits, phone check-ins, or telehealth sessions to monitor progress, offer reinforcement, and make adjustments as needed. Positive reinforcement for small achievements helps maintain momentum and engagement in the rehabilitation process.

Stroke prevention and nursing education

Stroke prevention is an essential aspect in handling the graveness of cerebrovascular disease, and the nurses have a role in primary and secondary prevention through education and health promotion [24]. Being frontline healthcare personnel, they can easily identify risk factors, promote healthy lifestyle changes, and deliver education targeted at patients, families, and communities. 

Primary prevention concentrates on ameliorating modifiable risk factors such as hypertension, diabetes, hyperlipidemia, smoking, physical inactivity, and poor diet. Nurses assess these risk factors while providing routine nursing care and counsel patients on lifestyle changes, establishment of habits for taking medications and screenings using evidence-based approaches [25]. The nursing profession itself could be engaged in community outreach and educational campaigns to spread awareness and early recognition and management of risk conditions for preventing stroke [26].

In the case of secondary prevention, especially for an individual who has had a stroke or transient ischemic attack (TIA), nursing education is essential. Nurses educate patients about antiplatelet or anticoagulant drugs, controlling hypertension, avoiding smoking, and modifying their diets [27]. They also help patients adhere to further follow-up care and rehab programs. Nurses are also taught to educate patients and their caregivers on early warning symptoms of stroke, in the use of FAST (Face, Arms, Speech, Time) acronym to recognize signs quickly. Prompt recognition and action can significantly reduce the severity of recurrent strokes and improve outcomes, making nursing education a cornerstone of secondary prevention strategies [28].

The Impact of Nurse-Driven Patient Education on the Prevention of a Secondary Stroke

Thus, the teaching that nurses provide patients ensures the prevention of secondary strokes by educating them and giving them the skills to carry out appropriate health management. Nurses further teach patients on appropriate control of risk factors such as hypertension, diabetes, high cholesterol levels, and medication adherence while discussing smoking cessation and heart-healthy lifestyle choices with them. Nurses support the prevention of recurrent strokes through patient engagement and providing motivation for behavioral change. Follow-ups and ongoing support help patients enhance their understanding and adhere to preventive measures over a long period, leading to improved health outcomes and fewer hospitalizations [29].

Review of the Lifestyle Modification Interventions Delivered by Nurses and Their Effectiveness in Stroke Prevention

In secondary stroke prevention and risk reduction, lifestyle modification interventions, with nurses as primary agents, have proven effective. Generally, these interventions address modifiable and immediate risk factors such as hypertension, smoking, lack of exercise, unhealthy diet, increased BMI, and excessive consumption of alcohol. Nurses assess risk from a patient perspective, counsel patients, and collectively set realistic goals that patients can achieve themselves. They educate the patient and provide ongoing support in ensuring that the patient maintains healthier choices, including but not restricted to physical activity, lowering salt and fat intake in nutrition, quitting smoking, and managing stress. 

The rationale behind such approaches is to promote behavioral change among patients, assisted by motivational interviewing and goal setting, further strengthening their motivation and commitment to these healthy behaviors. Some studies reveal that nurse-led lifestyle interventions improve patients' blood pressure and help them maintain ideal weight, and better cardiovascular conditions that can prevent primary and secondary strokes significantly [30]. Regular follow-ups and further community programs help strengthen the lifestyle changes and keep the momentum strong.

Management of post-stroke psychological consequences

Psychological consequences are quite common after a stroke, with depression and anxiety often interfering with recovery and life satisfaction [31]. Therefore, treatment of these disorders should be included in the total management of stroke, and nurses are crucial in the recognition, management, and support of patients with these conditions. Usually, the nurses are the first ones to notice the early signs of distress in a patient through their interactions. Early screening helps recognize early mood disorders or cognitive changes in patients if validated screening tools are used. Until the assessments end, nurses provide emotional support and counseling, and they can consider referring patients to mental health professionals or involving family members in care planning for a stronger support system.

Nurses provide education and reassurance, and help patients understand that psychological symptoms often occur and they can be treated. They also encourage patients to join support groups and participate in rehabilitative activities that foster social engagement and help decrease the feelings of loneliness. Nonpharmacological interventions, such as cognitive-behavioral techniques, relaxation training, and structured daily programming, might be used by nurses in collaboration with the multidisciplinary team [32]. They review the efficacy and side effects of medications prescribed for depression or anxiety to ensure their implementation is safe and well-coordinated. In brief, nurses have a very important role in the care of psychological aftereffects of stroke or TIA, helping patients cope with emotional challenges such as depression, anxiety, frustration, and post-stroke fatigue. They provide emotional support, connect patients with counseling or psychiatric services when needed, and encourage participation in support groups, all of which contribute to holistic recovery and improved quality of life.

*Nurse-Led Interventions in* *the* *Assessment and Management of Post-Stroke Depression and Anxiety*

Depression and anxiety represent some of the common psychological complications after a stroke, severely influencing recovery and rehabilitation engagement and basically the patient's quality of life. Nurse-level interventions hold great promise in assessing and managing these conditions through early detection, supportive care, and structured follow-up. In the setting of routine nursing care, nurses conduct assessments for the detection of mood and anxiety disturbances through validated screening tools like the Patient Health Questionnaire (PHQ-9) and the Generalized Anxiety Disorder (GAD-7) [33]. Early detection ensures immediate intervention, laying the foundation to the prevention of chronic long-term psychological distress.

Developing evidence from other recent studies further suggests the beneficial impact of nurse-led psychosocial interventions in managing depression and anxiety in patients with stroke. Interventions that included nurse counseling, psychoeducation, and routine follow-up significantly decreased depression and anxiety scores between weeks 0 and 12 in patients with stroke in a 2022 randomized controlled trial published in BMC Nursing [34]. Another 2022 study found that structured nurse-led telephone support with behavioral activation significantly improved emotional well-being and reduced hospital readmissions for mental health reasons [35]. 

The latter interventions usually include patient and caregiver education; goal setting; provision of emotional support through active listening, validation, and validation skills; and facilitation of referrals to mental health professionals when appropriate. Establishing a trusting relationship with nurses enables patients to develop effective coping strategies for managing psychological and social challenges, thereby fostering resilience and emotional stability. 

Review of Nursing Strategies Addressing Post-Stroke Cognitive Impairment and Caregiver Burden 

Cognitive impairment is a frequent symptom after stroke, arising as memory loss, attention deficits, or problems with problem-solving and communication [36]. These constitute barriers to a patient’s independence and, in turn, place a heavy burden upon caregivers. Nurses are at the forefront in combating such cognitive deficits and indeed caregivers through evidence-based and targeted strategies. Nursing interventions for cognitive impairment revolve around early assessment include using tools such as the Montreal Cognitive Assessment (MoCA) and ongoing monitoring throughout recovery [37]. Nurses engage patients in cognitive stimulation activities, encourage occupational therapy, and maintain structured daily activities conducive to cognitive functioning and adaptation. Education in cognitive changes equips patients and families to set realistic expectations and come up with strategies to cope. In parallel, the nurse addresses caregiver burden through emotional support, training, and education with regard to patient care responsibilities.

Nurses also inform caregivers about stroke-related cognitive changes and managing difficult behaviors, and stress on the importance of caring for themselves. Other nursing activities include facilitating support groups, arranging respite care, and making referrals to community agencies [38].

Studies have shown that nurse-led interventions, including structured follow-up and caregiver training programs, significantly reduce caregiver stress and improve confidence in managing post-stroke challenges [39]. A holistic approach that includes both patient and caregiver ensures better long-term outcomes for the stroke survivor and sustains the well-being of those providing care.

Stroke and communication disorders

Communication disorders, particularly dysphasia (or aphasia), are common after a stroke, especially when the brain’s language centers are affected [40]. These disorders can severely impact a patient's ability to express needs, understand others, and participate in rehabilitation, ultimately affecting quality of life and recovery. Nursing care plays a vital role in supporting communication, managing frustration, and fostering an environment that encourages engagement and adaptation.

Review of Nursing Interventions for Managing Dysphasia and Communication Deficits Post-Stroke

Research shows that nursing interventions can significantly support patients with dysphasia and other communication deficits. Nurses are often the primary point of contact for stroke survivors and are in a unique position to implement communication supportive strategies. Interventions include the use of non-verbal communication tools, simplified language, written cues, picture boards, and consistent routines to facilitate understanding. Studies have emphasized the importance of early recognition of communication issues and integrating speech-language therapy into the care plan [41].

Recent literature, including studies published around 2021-2022, supports nurses' involvement in multidisciplinary communication programs [42]. For example, a study in the Rehabilitation Nursing (2021) found that training nurses in supported conversation techniques significantly improved patient interaction and satisfaction [43]. Another study highlighted the effectiveness of nurse-led communication screenings to facilitate early referrals to speech therapists, leading to better language recovery outcomes [44].

Effectiveness of Specialized Nursing Care in Improving Communication Outcomes in Stroke Patients

Specialized nursing care tailored to communication needs has been shown to enhance recovery and patient engagement. Nurses trained in speech-supportive techniques can help reduce patient frustration, improve mood, and encourage participation in other aspects of rehabilitation. They play a crucial role in ensuring consistency in communication methods across care settings, reinforcing speech therapy goals during routine interactions.

Evidence also shows that when nurses are trained to use augmentative and alternative communication (AAC) tools and to apply individualized communication plans, patients demonstrate improved ability to express needs and participate more actively in care decisions [45]. These interventions not only support the patient but also reduce caregiver stress by improving understanding and reducing communication-related anxiety.

Technological advances in stroke care

Technological innovations have transformed stroke care, enabling faster diagnosis, improved treatment coordination, and better long-term monitoring. Nurses are increasingly integrating digital tools into patient care, especially in the acute and post-acute phases. Technologies such as telemedicine, tele-nursing, and mobile health applications are enhancing the accessibility, efficiency, and quality of stroke management [46].

Impact of Telemedicine and Tele-Nursing in Stroke Management and Patient Outcomes

Telemedicine has become a critical component in stroke care, particularly for patients in remote or underserved areas. It allows real-time consultations with stroke specialists, enabling faster decision-making for interventions like thrombolysis. Tele-nursing complements this by extending nursing support through virtual platforms, offering education, monitoring, and follow-up care beyond the hospital setting [47].

Studies from 2022 highlight the effectiveness of tele-nursing in improving continuity of care and patient satisfaction [48]. For instance, a study published in Stroke (2022) demonstrated that patients who received nurse-led telehealth follow-up after discharge showed higher medication adherence and fewer readmissions [49]. Tele-nursing also helps monitor for early signs of complications, ensuring timely intervention, and reducing the burden on emergency services.

Review of Mobile Health Applications Utilized by Nurses to Enhance Stroke Care Delivery and Monitoring

Mobile health (mHealth) applications are increasingly used by nurses to support stroke care. These apps assist in tracking vital signs, medication adherence, rehabilitation exercises, and symptom reporting. They also serve as educational tools for both patients and caregivers. Nurses use mHealth platforms to remotely monitor patient progress, send reminders, and provide real-time feedback. For example, apps designed for stroke rehabilitation can guide patients through personalized exercises, with nurses reviewing performance data and adjusting care plans accordingly. A 2022 review in Journal of Medical Internet Research (JMIR) Nursing found that mobile apps used in post-stroke care improved patient engagement, enhanced self-management, and facilitated timely communication between nurses and patients [50]. 

Stroke care in vulnerable populations

Vulnerable populations, such as racial and ethnic minorities, low-income individuals, older adults, and those in rural areas, often face significant disparities in stroke care. These disparities may stem from limited access to healthcare, socioeconomic challenges, and systemic inequities. Nurses play a key role in identifying and addressing these gaps through tailored interventions and culturally competent care.

Literature Review Exploring Disparities in Stroke Care Delivery and Outcomes, and Nursing Interventions Aimed at Reducing These Gaps

Research consistently shows that vulnerable groups are less likely to receive timely stroke treatment, such as thrombolytic therapy, and often have poorer outcomes, including higher mortality and disability rates. A review of studies between 2020 and 2022 revealed persistent disparities in stroke recognition, emergency response, and rehabilitation access, particularly among Black, Hispanic, and rural populations [51].

Nurses are actively working to close these gaps through community education, outreach programs, and individualized care planning. Nurse-led initiatives, such as stroke awareness campaigns in underserved communities, have been shown to improve early recognition of symptoms and encourage quicker access to emergency care. In clinical settings, nurses advocate for equal treatment access, provide discharge planning tailored to patients’ socioeconomic contexts, and coordinate with social services to ensure continuity of care [52].

Cultural Competence and Nursing Care Strategies for Stroke Patients from Diverse Populations

Culturally competent nursing care is essential in providing effective, respectful, and responsive care to patients from diverse backgrounds [53]. It involves understanding the beliefs, values, language, and health practices of the patients, and integrating that knowledge into the care plan.

Nurses implement culturally sensitive strategies by using interpreter services, incorporating culturally relevant education materials, and involving family members in decision-making when appropriate. Cultural competence training for nurses has been linked to improved patient satisfaction, better communication, and increased adherence to care and rehabilitation plans. For example, culturally-tailored stroke education programs led by nurses significantly improved knowledge retention and engagement among minority stroke survivors [54]. These strategies help build trust and empower patients, ultimately improving outcomes and reducing disparities.

Nutritional management post-stroke

Nutritional management is vital in post-stroke care, for many patients face problems with eating and maintaining adequate nourishment. Some of the common problems include dysphagia, malnutrition, and dehydration. These must be dealt with immediately because they might prolong patient recovery, increase the risk of further complications, and lengthen the hospital stay. Nurses have a key role in the identification and management of such problems through an in-depth assessment, intervention into the problem area, and support for the patient [55].

Review of Nursing Interventions Seen in Dysphagia, Malnutrition, and Hydration-Related Problems Post-Stroke

Dysphagia, or difficulty in swallowing, is a common problem following stroke and places the patient at risk of aspiration pneumonia and malnourishment. Nurses ensure early screening for swallowing difficulties, often by conducting bedside assessments and referring to speech and swallowing specialists. Following these assessments, nurses implement dietary modifications, such as providing texture-modified foods and thickened fluids, to allow for safe consumption. In order to prevent malnutrition and dehydration, nurses observe food and fluid intake, look out for signs of declining nutrition, and work closely with dieticians to adjust diets as needed [56]. They may also advocate for enteral nutrition should oral feeding become inadequate, reminding one that such interventions are temporary and will help the patient recover fully from the stroke.

Nursing Role in Improving Nutritional Outcomes Through Assessment, Monitoring, and Education in Stroke Patients

Nurses play a proactive role in improving nutritional outcomes through routine assessments and individualized care planning. This involves evaluating the nutritional status of patients upon admission and throughout hospitalization using standardized tools [57]. Nurses also track weight changes, monitor lab values, and assess signs of dehydration to detect issues early. Education is another key aspect of nursing care. Nurses provide guidance to patients and caregivers on appropriate dietary choices, safe swallowing techniques, and the importance of hydration in recovery. They also offer support in managing feeding-related anxieties or frustrations, which are common in patients with swallowing difficulties [58].

Quality improvement in stroke nursing care

Quality improvement in stroke nursing care focuses on enhancing clinical outcomes, patient safety, and care efficiency through systematic, evidence-based approaches. Nurses are integral to these efforts, leading initiatives that refine protocols, streamline care processes, and foster a culture of continuous improvement within stroke care units.

Evidence-Based Nursing Practices in Stroke Care Units and Their Impact on Patient Outcomes and Hospital Readmissions

The integration of evidence-based nursing practices in stroke care units has significantly improved patient outcomes. Standardized protocols for early stroke recognition, timely intervention, monitoring for complications, and adherence to rehabilitation pathways have contributed to reduced mortality, improved functional recovery, and fewer hospital readmissions [59]. Nurses implement and maintain these practices by following clinical guidelines, participating in multidisciplinary stroke teams, and using tools such as care bundles and checklists. Continuous education and training ensure that nursing interventions remain aligned with current best practices, promoting consistent and high-quality care delivery [60].

Review of Nursing-Led Quality Improvement Projects Aimed at Enhancing Stroke Care Standards

Nurses frequently lead quality improvement (QI) projects that target specific challenges within stroke care [61]. These projects often focus on improving areas such as early dysphagia screening, time-to-thrombolysis, patient education, post-discharge follow-up, and prevention of complications like pressure ulcers or infections.

By collecting and analyzing data, setting measurable goals, and implementing targeted interventions, nursing-led QI initiatives drive meaningful change. These projects not only improve clinical processes but also empower nurses to take active roles in advancing care standards. Many initiatives result in sustained improvements in patient satisfaction, care coordination, and efficiency within stroke units [62].

Family and caregiver support in stroke nursing

Family members and caregivers play a vital role in the recovery and long-term care of stroke survivors. However, the sudden onset and complex nature of stroke can leave caregivers unprepared, overwhelmed, and at risk of emotional, physical, and financial strain. Nurses are essential in supporting both the patient and their caregivers by providing guidance, education, and emotional support throughout the continuum of care. Nurses begin caregiver support early during the hospital stay by involving families in care planning and education. This includes teaching them about the effects of stroke, potential complications, medication management, mobility assistance, and rehabilitation processes. Clear communication helps caregivers understand the patient’s condition and anticipate the level of support needed after discharge [63].

Beyond education, nurses provide emotional support by actively listening to concerns of the caregivers, validating their feelings, and connecting them with counseling or peer support services when needed. In cases of caregiver burnout or high stress, nurses may also advocate for respite care or involve social workers to explore additional resources. In post-discharge settings, nurses maintain communication with families through follow-up calls or home visits, ensuring caregivers feel supported and confident in managing the patient’s needs. They also promote caregiver well-being by encouraging self-care practices and regular health check-ins [64].

Impact of Nursing Interventions on the Well-being of Caregivers of Stroke Patients

Nursing interventions significantly influence the emotional, physical, and psychological well-being of caregivers. Caregivers often experience high levels of stress, anxiety, and fatigue as they adjust to the demands of supporting a stroke survivor. Nurse-led interventions, such as structured education, emotional support, and regular follow-up, help reduce caregiver burden and improve coping capacity [65].

By providing clear guidance on caregiving tasks, early training, and resources for stress management, nurses enhance the confidence of caregivers and reduce their uncertainty. Supportive nursing practices also promote better mental health outcomes among caregivers, helping them sustain their caregiving role without compromising their own well-being [66].

Review of Effective Nursing Strategies for Caregiver Education, Support, and Empowerment Post-Stroke

A review of recent literature reveals several effective nursing strategies for educating, supporting, and empowering caregivers of patients with a stroke [67]. These include individualized education plans that address specific caregiving needs, hands-on training sessions before discharge, and the use of written materials and digital tools to reinforce learning.

Support interventions, such as counseling, peer support groups facilitated by nurses, and regular check-ins, have been shown to reduce emotional distress and improve caregivers' quality of life [68]. Empowerment-focused strategies, such as involving caregivers in goal-setting and decision-making, enhance their sense of control and preparedness. Collectively, these nursing approaches not only support caregiver well-being but also contribute to improved patient outcomes, as well-supported caregivers are more effective in managing the stroke survivor’s recovery and preventing complications.

## Conclusions

Acute stroke management is a complex and time-sensitive process that demands a multidisciplinary approach, with nurses playing a pivotal role across every phase of care. From the hyperacute stage, where rapid assessment and coordination for interventions like thrombolytic therapy are critical, to the recovery and rehabilitation phases, nursing interventions significantly influence patient outcomes. Nurses are not only involved in clinical care, such as monitoring vital signs, managing complications, and assisting with mobility, but also in providing emotional support, educating patients and families, and facilitating adherence to treatment and rehabilitation programs.

Throughout the continuum of stroke care, evidence-based nursing practices contribute to reduced mortality, fewer hospital readmissions, and improved functional recovery. Nurse-led interventions in areas such as nutritional support, psychological care, and communication management address the holistic needs of stroke patients, enhancing both their physical and mental well-being. Additionally, nurses serve as key advocates for vulnerable populations, working to close care gaps and deliver culturally competent, equitable services.
